# Lake Superior's summer cooling of shorelines and adjacent inland forests: Implications for refugia of boreal forests and disjunct arctic–alpine plants

**DOI:** 10.1002/ece3.10833

**Published:** 2023-12-28

**Authors:** Ashley Hillman, Scott E. Nielsen

**Affiliations:** ^1^ Department of Renewable Resources University of Alberta Edmonton Alberta Canada

**Keywords:** climate refugia, Lake effect, Lake Superior, vegetation transitions

## Abstract

Climate refugia can serve as remnant habitat for cold‐adapted species and delay forest transitions. The world's largest freshwater lake by surface area, Lake Superior, serves as a model system for understanding cooling‐mediated refugia effects, as its cool summer water temperatures have maintained disjunct populations of arctic–alpine plants on its shoreline since deglaciation. It is known to affect local inland climates by providing a summer cooling effect; however, the inland temperature gradient and spatial patterns of cooling have not been well quantified. Here, we describe the extent, degree, and patterns of temperature buffering and examine drivers of buffering and disjunct plant occurrence for Lake Superior's north shore over a 3‐year period at distances of 10, 100 m, 1, 10, and 100 km inland. We analyzed temperature data by year, month, summer maximum (July), and growing degree days (GDD_0_) for each site. Average summertime cooling at shore sites (10 m) was ~5°C cooler than reference sites (100 km inland), with a maximum difference of −19.2°C. The magnitude of cooling varied geographically, with sites further west and southeast showing little to no cooling effect, while the exposed north‐central shore showed the highest degree of buffering (5.8°C cooler) and had a shorter growing season than reference sites. Finally, north‐central shorelines had fewer days above 16°C, a threshold above which disjunct plants are unlikely to grow. These sites also showed the highest proportion of disjunct arctic–alpine species, reflecting the highest buffering from inland sites. On north‐central shores, sites up to 10 km inland had less than 10 days per year warmer than 20°C, a threshold identified for boreal forest transition. An understanding of the extent of lake‐mediated cooling on adjacent forests can better inform the risk to disjunct species, inland forests, and vegetation transition models on Lake Superior's north shore.

## Graphical abstract

Large lakes can provide a climate refugia effect on their shorelines and adjacent inland forests, providing potential buffering from regional vegetation transitions. Here, we describe the extent, degree, and patterns of temperature buffering for Lake Superior's north shore, with implications for cold‐adapted plants on the shoreline and adjacent inland regions.

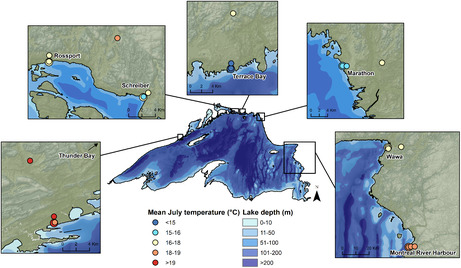

## 1 INTRODUCTION

As the climate warms, it is important to identify areas that remain buffered from warming temperatures. Cold‐adapted species must either migrate or adapt to changing climates (Chen et al., 2011). However, the current rate of climate warming makes either option less likely, and thus the identification of places that lag or buffer climate change is of high conservation value. The term climate refugia has been used to describe areas of relatively stable climate during global climate shifts (Bennett & Provan, 2008; Stewart et al., 2010), serving as areas where species persist and as nuclei for dispersal. The most recent large‐scale example of refugia‐mediated recolonization in North America is the period after the last glacial maximum, in which species persisting in refugia south of the ice sheets were able to recolonize as the glaciers retreated (Bennett & Provan, 2008). More recently, however, the term climate refugia has been used in conservation to describe areas that are likely to maintain a relatively stable climate during the current climate warming crisis (Ashcroft, 2010; Barnosky, 2008; Stralberg et al., 2020). Here, refugia are identified as places that buffer or decouple the local climate from that of the regional climate (De Frenne et al., 2021; Dobrowski, 2011). Buffering is a dampening of macroclimate, so macroclimate fluctuations are experienced by the localized microclimate to a lesser degree (De Frenne et al., 2021). Decoupling, however, refers to the complete independence of macroclimate and microclimate so that one does not influence the other (De Frenne et al., 2021). This refugia effect can occur across a variety of scales, affecting a variety of organisms and processes (Balantic et al., 2021; Baumgartner et al., 2018; Billman et al., 2021; White et al., 2022), and can be influenced by a number of landscape characteristics. Topographic effects, such as mountains and hill systems, create elevational gradients in temperature, with higher elevations, topographic depressions, and north‐facing slopes generally being cooler (Estevo et al., 2022; Scherrer & Körner, 2011). Regional to local hydrologic patterns can also drive microclimates and thus refugia (McLaughlin et al., 2017), with springs (Cartwright et al., 2020), wetlands, lakes, and peatland complexes providing buffering effects of climate change, including wildfire (Nielsen et al., 2016; Stralberg et al., 2020). Lakes can also provide a cooling effect on their shorelines, dubbed the “lake effect” (Hinkel & Nelson, 2012). Upwellings of cold water are brought to the surface during the lake's annual cycling regimes, and winds push these cooler temperatures inland (Changnon & Jones, 1972). The scale of this lake effect varies, but large, deep lakes can provide a cooling effect that extends many kilometers inland.

The Great Lakes region of eastern Canada/United States is known to have a strong lake effect, influencing climate up to 80 km from its shore (Scott & Huff, 1996). Not surprisingly, the largest of the Great Lakes, Lake Superior, also has the greatest lake effect. The largest lake in the world by surface area, Lake Superior's size and depth buffer its shorelines from the surrounding climate, with temperature differentials up to 6°C cooler in summer and 2°C warmer in winter (Changnon & Jones, 1972; Scott & Huff, 1996). This lake effect is strong enough that the shoreline contains microclimates that support populations of arctic and alpine plants that have persisted since deglaciation, resulting in disjunct biogeographic patterns (Given & Soper, 1981). These species include *Saxifraga oppositifolia*, *Dryas integrifolia*, *Sagina nodosa*, and *Vaccinium uliginosum* (Given & Soper, 1981), all typically found in high alpine and arctic environments. Separated by ~500 km on average from their core range, populations at Lake Superior are restricted to a narrow band of exposed bedrock shoreline. Species observed here are typical of high alpine and tundra environments in that they are slow‐growing, have a small stature, and predominantly reproduce vegetatively (Grime, 1977). Dispersal out of the Lake Superior region is unlikely due to hundreds of kilometers of unsuitable habitat between populations and the species' poor dispersal rates (typically ~1 m from the parent plant; Vittoz & Engler, 2007); therefore, they are reliant on the maintenance of cool shorelines to persist. However, shoreline summer cooling is not consistent across the lake, as patterns of wind exposure and topographic barriers, such as islands, can influence local temperatures (Hinkel & Nelson, 2012). In general, wind direction is west‐northwest, and thus east and northeastern shores receive more wind and cooler temperatures as the wind crosses the deepest parts of the lake. Similarly, offshore islands and peninsulas can reduce the wind exposure of other shorelines, resulting in warmer summer temperatures. The geography of the lake and shoreline can thus affect the extent and magnitude of summer cooling exhibited by the lake. This can subsequently affect local patterns in vegetation, including disjunct arctic–alpine flora.

Patterns and magnitudes of cooling can also affect plants further inland from the exposed bedrock shoreline. The north shore of Lake Superior serves as a transition zone between deciduous‐dominated hardwood forests on the south shores and conifer‐dominated boreal forests in the north. Warmer temperatures are projected to shift forest zones north in the Great Lakes regions, resulting in an overall decline in conifer species (Walker et al., 2002) as they are replaced by deciduous trees that are better adapted to warm conditions. Summer temperatures are thought to be affecting transitions in forest regeneration (Fisichelli et al., 2014), with boreal forests transitioning to deciduous‐dominated forests when summer temperatures are warmer than ~20°C (Frelich et al., 2021; Scheffer et al., 2012). Lake‐mediated cooling of adjacent inland forests could play a role in slowing the transition to deciduous‐dominated forests, providing refuge for economically and ecologically important conifer trees.

Understanding the extent of shoreline summer cooling is important, particularly since Lake Superior's water temperatures, which are a main driver of shoreline temperatures and disjunct arctic–alpine plant presence (Hillman & Nielsen, 2023), are increasing at a faster rate than atmospheric temperatures (Austin & Colman, 2007). Despite the summer surface water temperature of Lake Superior being cold (~16°C on average, but can be as low as 8°C on some parts of the lake; Bennett, 1978), summer surface water temperature has increased ~3.5°C over the last century, and mostly over the last several decades (Austin & Colman, 2008). Ice consistently forms along the shorelines and bays of the lake each winter (Bennington et al., 2010), but there is a general trend toward less winter ice cover and, in particular, a shorter winter ice season that has contributed to the recent warming of the lake (Austin & Colman, 2008). Indeed, in the winter of 2023, record lows for ice cover were observed for the Great Lakes, with Lake Superior having just 6% ice cover in March (down from the 39% annual average; NOAA, 2023). While offshore winds can contribute to the cooling of shorelines, especially in areas of consistent upwellings (Plattner et al., 2006), increasing summer surface water temperatures may continue to have a warming effect on Lake Superior's shoreline and adjacent forests.

While the general regional lake effect of Lake Superior is well known, localized effects at the shoreline and adjacent forests have not been fully quantified. Remote sensing data capture regional trends in climate around the lake but are at too coarse of a scale to capture local microclimatic effects of the shore itself, where disjunct arctic–alpine plants occur. Weather stations are not common in the area, with stations being located in towns away from the coastline (up to 10 km inland), and thus temperature data on the shoreline and adjacent forests is lacking. Discrepancies between regional weather stations and localized microclimate could de‐emphasize temperature buffering, and an understanding of the degree of temperature difference between regional and localized climate could better inform climate models. Some work has been done to quantify this discrepancy; however, few studies have been conducted on Lake Superior inland temperature gradients. In one study, Hinkel and Nelson (2012) found that nearshore temperatures were 1–2°C colder than areas 5 km inland. However, these sites were on the south shore, where the lake is shallow, and only in a single localized area. The north shore of Lake Superior is known to have much cooler summer temperatures, more shoreline exposed to deep water, and thus higher refugia potential (Hillman & Nielsen, 2023). However, little has been done to quantify the extent and magnitude of the lake effect on the north shore of Lake Superior, where it is expected to be most pronounced. Our aim here is to address this gap by quantifying, for the north shore of Lake Superior, the extent of the inland cooling gradient, the degree of buffering from inland temperatures, and patterns of cooling. We predict that the more exposed north and northeast shores will have the greatest temperature buffering, and that cooling will extend at least 1 km inland. Understanding these patterns helps inform the current and future refugia potential of the Lake Superior shoreline and thus its relationship to cold‐adapted plant species.

## 2 METHODS

### 2.1 Study area

**FIGURE 1:**
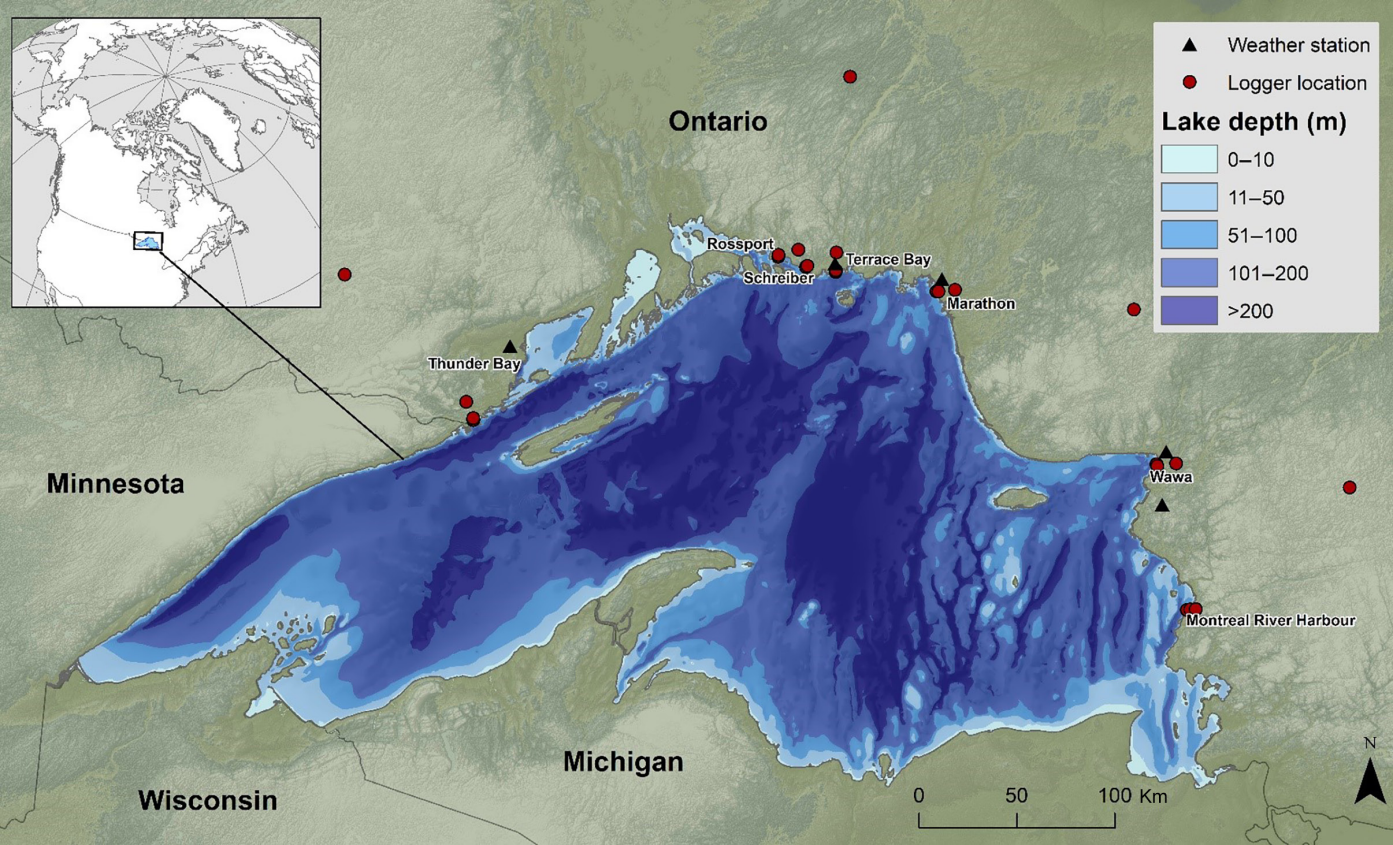
Study area at Lake Superior and location of temperature loggers. The lake is 563 km east to west and 258 km south to north, with a maximum depth of 406 m. The lake is bordered by Ontario in Canada in the north, and in the south by Wisconsin, Minnesota, and Michigan in the United States (see inset map). Locations for 31 temperature loggers are denoted with a red circle, while locations of the nearest meteorological station are denoted with a black triangle.

Our study area is located on the north shore of Lake Superior, in Ontario, Canada (Figure 1). Lake Superior is the largest freshwater lake in the world by surface area (82,103 km^2^), stretching 563 km east to west and 258 km south to north (Bennington et al., 2010) and following the axis of the North American continental rift (Ojakangas et al., 2001). The average water depth is 147 m, with a maximum depth of 406 m. The mean annual temperature of the lake is 4.5°C (Hinkel & Nelson, 2012).

The study area is considered part of the Boreal Shield ecozone of Canada (Ecological Stratification Working Group, 1995). This region is typified by Precambrian bedrock, consisting primarily of granite, gneiss, and volcanic rock (Crins et al., 2009). The majority of the landcover consists of mixed coniferous and deciduous forest cover, with black spruce (*Picea mariana*), white spruce (*Picea glauca*), balsam fir (*Abies balsamea*), trembling aspen (*Populus tremuloides*), paper birch (*Betula papyrifera*), jack pine (*Pinus banksiana*), and white cedar (*Thuja occidentalis*) as the dominant trees, with eastern white pine (*Pinus strobus*) and red pine (*Pinus resinosa*) less common and absent in the far northern parts of the lake.

### 2.2 Field study design

Five temperature logger transects were established in July 2018 at Thunder Bay, Terrace Bay, Marathon, Wawa, and Montreal River Harbour (Figure 1). An additional two transects were established in July 2019 at Rossport and Schreiber (Figure 1). Due to minimal access to shoreline and inland locations throughout much of the study area, transects are not evenly distributed along the shore but are located in areas that were accessible by road. Temperature station locations along transects were on a logarithmic scale moving inland from the lake, with loggers established at 10, 100 m, 1, 10, and 100 km. Loggers at 100 km were considered reference sites, as we expected lake effects to be unlikely at this distance. Some were also directly north of the lake, where latitudinal cooling (not warming) would be expected. Along each transect, we used HOBO loggers (model UA‐002‐64, manufactured by Onset, Pocasset, MA) placed inside a radiation shield (Holden et al., 2013) and attached to the north side of a tree at a standard height of 1.3 m. Efforts were made to select sites that were within a similar canopy closure with similar dominant tree species to reduce temperature change from localized site conditions. At each logger station, we established a 10 m × 10 m plot with the logger as the center. Canopy closure was measured using a concave spherical densiometer adjacent to the tree the logger was placed on to remove the canopy influence of the tree itself. We took an average of four measurements each (facing each cardinal direction) at the center and four corners of the plot, and then an overall site average to get a better representation of site canopy closure. We also noted dominant tree species within the plot. As Rossport, Schrieber, and Terrace Bay are in close proximity to each other and inland access is limited, these three transects share a reference logger, and Rossport and Schreiber share a 10 km logger. Similarly, Wawa and Montreal River Harbour share a reference logger due to accessibility limitations. Loggers were programmed to record temperatures every 30 min.

On the shoreline adjacent to each 10 m logger site, we conducted a survey of disjunct arctic–alpine plant richness. We established a 10 m × 10 m plot and subsampled three microhabitat (bedrock cracks and splash pools) locations to determine the presence, abundance, and richness of disjunct species. At the microhabitat surveyed, a full species list was compiled for both disjunct species and common boreal species, and we counted individual stems of each species to determine abundance. Shore width was also measured at each site, determined as the distance in meters from the edge of the water to the edge of continuous tree cover.

### 2.3 Statistical analyses

We summarized temperature data for the period between July 13, 2018, and July 31, 2020 for Thunder Bay, Terrace Bay, Marathon, Wawa, and Montreal River Harbour, and between July 13, 2019 and July 31, 2020 for Rossport and Schreiber. Refer to Table A1 in Appendix 1 for dates of individual logger data. The mean temperature across distances was compared using a two‐sample *t*‐test, with emphasis on July daytime temperatures (6:00 sunrise to 22:00 sunset) as these reflect the highest refugia potential (maximum summer temperatures). The mean July daily temperature was compared between each logger and the nearest meteorological station (Environment and Climate Change Canada, 2023), using a two‐sample *t*‐test. The nearest meteorological stations varied from 3.75 km (Marathon) to 10.27 km (Montreal River Harbour) inland from the shoreline (Figure 1).

We modeled environmental drivers of temperature difference between loggers using linear mixed‐effect models in the glmmTMB package in R (Brooks et al., 2017), setting transect as a random effect to account for the nested design of our sites. Elevation data were acquired from the United States Geological Survey's Shuttle Radar Topography Mission's 30 m digital elevation model (United States Geological Survey, 2009), with lake surface subtracted from shoreline elevation to measure elevation above the lake's surface. We acquired geospatial data from the National Oceanic and Atmospheric Administration on bathymetric data for lake depth (NOAA (1), 2020) and average annual and July water surface temperatures (2002–2013, NOAA (3), 2020). Because the effects of these lake variables on inland conditions may vary by scale, four different overlapping windows were examined, ranging from near shoreline (0–1 km or 0–5 km) to far from shoreline (0–10 km or 0–50 km) scales. We used shore width measured from shoreline field plots as a proxy for exposure, as high winds and wave action associated with high fetch across the lake generally batter more exposed areas of the lake, resulting in a wider section of exposed bedrock. All predictor variables were assessed for collinearity using Pearson correlation tests. Variables with a correlation *r* > |.7| were considered colinear and not included within the same model structure. We used a purposeful selection of covariates (Bursac et al., 2008) for model selection.

To observe the effects of temperature difference on potential plant growth, we used growing degree days (GDD) and average days above a base temperature threshold as proxies for effective growing seasons and plant transition thresholds. GDD is a measure of the accumulated heat units above which plant development will occur and is an effective predictor of phenology (Quaglia et al., 2020). To compare GDD between sites, we calculated the cumulative sum of daily mean temperatures (Mcmaster & Wilhelm, 1997) above 0°C (GDD_0_) across the growing season. We used 0°C instead of the commonly used 5°C base threshold to account for the lower thermal requirements of arctic and alpine plants at shoreline locations (Quaglia et al., 2020). We considered the growing season to be April 1 to September 30, and thus 2019 was the only year with full growing season data for Thunder Bay, Terrace Bay, Marathon, Wawa, and Montreal River Harbour. As Rossport and Schreiber loggers were not established until July 2019, full growing season data were unavailable for 2019, and thus GDD_0_ was calculated for the 2020 growing season for these sites. GDD_0_ was not calculated for Montreal River Harbour's 10 m site, as a data logger malfunction resulted in the loss of part of the growing season's data. We set two separate base temperature thresholds to compare between sites. To determine patterns in the habitat availability of disjunct arctic–alpine plants, we first compared the average number of days per year above 16°C, a temperature threshold identified as the temperature at which most arctic–alpine plants are no longer predicted to occur on the Lake Superior shoreline (Hillman & Nielsen, 2023). Only shoreline sites were compared at the 16°C threshold, as disjunct arctic–alpine species do not occur at sites further inland. To better understand the potential for sites adjacent to the lake to resist forest transition, we compared the average number of days per year above 20°C, a temperature threshold above which conifer‐dominated forests begin to transition to deciduous forest (Frelich et al., 2021; Scheffer et al., 2012).

Additionally, we modeled drivers of the proportion of disjunct plants on adjacent shore sites using beta regression in the betareg package for R (Cribari‐Neto & Zeileis, 2010). We used coverage‐based rarefaction in the iNEXT online software (Chao et al., 2016) to confirm that observed richness represented the full species community at the site (Chao et al., 2014). We included measures of growing season as predictor variables, including GDD_0_, mean July daytime temperature, and the number of days above the 16°C threshold for disjunct arctic–alpine species presence. We also included shore width as a proxy for exposure. All predictor variables were assessed for collinearity using Pearson correlation tests. Variables with a correlation *r* > |.7| were considered colinear and not included within the same model structure. We used a purposeful selection of covariates (Bursac et al., 2008) for model selection. All analyses used R v.4.3.1 (R Core Team, 2022).

## 3 RESULTS

### 3.1 Field survey results

Canopy closure at the majority of sites ranged between 80% and 90%, reflecting similarities in conditions between sites. Only the 10 km site at Marathon and the 100 km site at Terrace Bay had canopy closures less than 80%, at 62% and 76%, respectively. The most common tree species at logger sites were *Abies balsamea*, *Picea glauca*, and *Betula papyrifera*. At most sites, *Abies balsamea* was the dominant tree, typically making up 25%–75% of the tree cover at the site. Elevation varied from a minimum of 4 m above the lake at shoreline sites to a maximum of 290 m above the lake at 100 km reference sites. Thunder Bay showed the largest difference in elevation between the shoreline site and reference sites, at 284 m. Schreiber and Terrace Bay had the smallest difference in elevation between shoreline sites and reference sites, at 188 m. Refer to Table A1 in Appendix 1 for complete site information for all sites.

Sites on the north‐central shore had the highest richness of disjunct species. Although observed species richness was low (three species), Rossport had the highest proportion of disjunct species, at 0.67. Schreiber had higher overall richness, at 10 species, and a proportion of disjunct species of 0.50. Terrace Bay showed the highest overall site richness, with 20 species, eight of which were disjunct species (proportion = 0.40). Sites on the east shore had a low proportion of disjuncts, with Wawa shoreline having three disjunct species from an overall site richness of 11 species. The Thunder Bay shoreline did not have any disjunct species. The most commonly recorded disjunct species across sites were *Pinguicula vulgaris*, *Primula mistassinica*, *Saxifraga paniculata*, *Trichophorum cespitosum*, and *Trisetum spicatum*. For a full list of disjunct species recorded at shoreline sites, refer to Table A2 in Appendix 1.

### 3.2 Temperature trends

**FIGURE 2:**
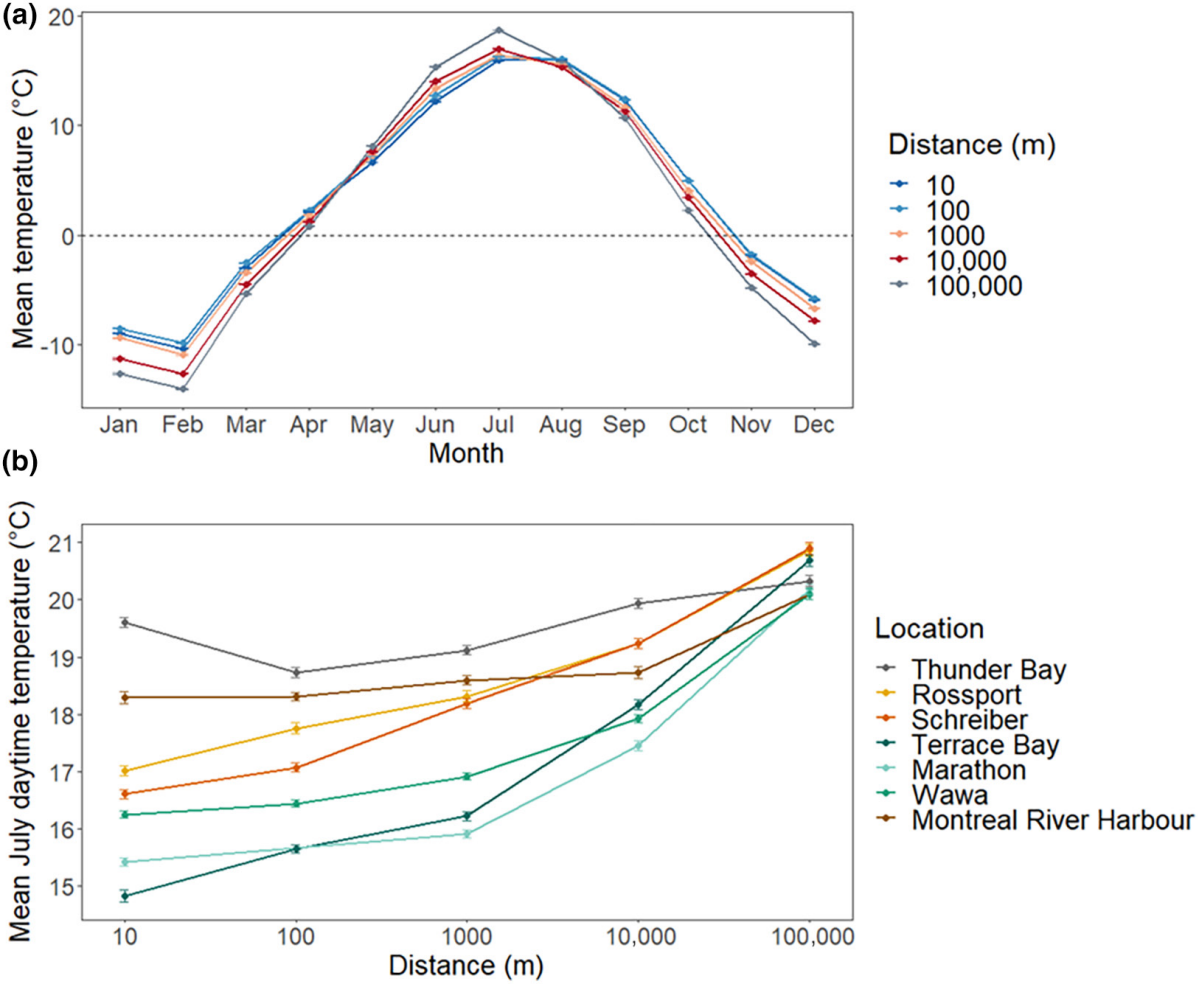
Mean annual and mean July daytime temperature trends. Mean annual temperature trends (a) show a shoreline warming effect in the winter months and a cooling effect up to 10 km inland in the summer months. Mean July daytime temperatures (b) vary by transect but can be up to 5.8°C cooler at a nearshore site than at control plots 100 km inland.

Lake effects were most pronounced at 10 and 100 m from the shore, but temperature differences were noted for some transects up to 10 km inland (maximum lakeshore distances) when compared to the 100 km reference site. The mean annual temperature did not significantly differ between sites, suggesting temperature buffering but not decoupling. Despite no significant difference in mean annual temperature, strong seasonal buffering effects were observed. Winter (December–February) average temperatures were buffered at −5.8 and −5.3°C at 10 and 100 m, respectively, compared with −9.0 and −10.6°C at 1 and 10 km, respectively (Figure 2a).

**FIGURE 3:**
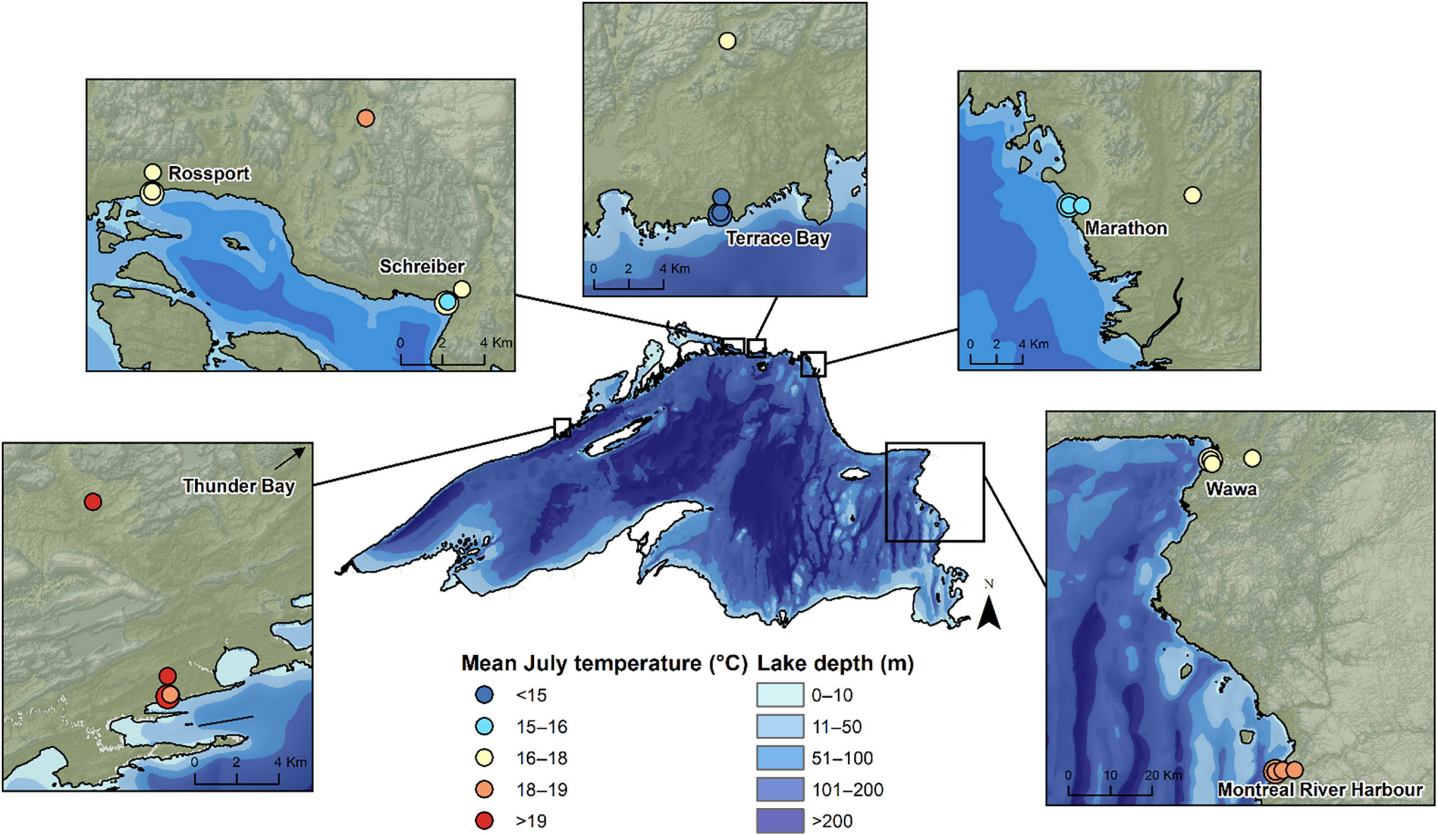
Mean July daytime temperature at shore and inland sites. Mean July daytime temperatures vary geographically, with sites on the north and northeast exposed shorelines showing lower temperatures than sites on the west and southeast shores.

Summer temperatures provide the largest cooling effect for cold‐adapted plant species; however, mean July daytime temperatures vary widely across transects (Figure 2b). Thunder Bay shows the lowest difference between sites and is generally the warmest transect. Marathon and Terrace Bay have the coldest shoreline temperatures, at 15.4 and 14.8°C, respectively. Terrace Bay also had the coldest July daytime temperatures along its entire transect. Montreal River Harbour, despite being on the highly exposed eastern shore, shows little temperature buffering, with all sites differing from the 100 km site by <1.8°C (Figure 3). Refer to Table 1 for a complete list of mean July daytime temperatures for each site.

**TABLE 1:** Summary of July daytime temperature for all sites.

Location	Distance inland	Mean July temp (SD) (°C)	Difference from 100 km (°C)	Difference from met station (°C)	GDD_0_	Days above 20°C
Thunder Bay	10 m	19.6 (4.7)	−0.7	−1.9	2163	17
	100 m	18.7 (4.2)	−1.6	−2.3	1976	12
	1 km	19.1 (3.9)	−1.2	−1.8	2043	17
	10 km	19.9 (4.5)	−0.4	−1.0	2230	29
	100 km	20.3 (5.2)	0.0	−0.8	2115	39
Rossport^a^	10 m	17.0 (4.0)	−3.8	−0.2	1939	5
	100 m	17.7 (4.4)	−3.1	0.2	2057	8
	1 km	18.3 (4.3)	−2.5	0.7	2042	7
	10 km	Same as Schreiber 10 km site				
	100 km	Same as Terrace Bay 100 km site				
Schreiber^a^	10 m	16.6 (3.4)	−4.3	−0.7	1875	1
	100 m	17.1 (3.6)	−3.8	−0.3	1752	3
	1 km	18.2 (3.7)	−2.7	0.6	2037	7
	10 km	19.2 (3.9)	−1.7	1.3	1963	9
	100 km	Same as Terrace Bay 100 km site				
Terrace Bay	10 m	14.8 (4.0)	−5.8	−1.1	1915	1
	100 m	15.7 (3.9)	−5.0	−0.9	1977	1
	1 km	16.2 (4.3)	−4.5	−0.9	1889	2
	10 km	18.2 (4.4)	−2.5	0.9	1974	9
	100 km	20.7 (5.0)	0.0	0.9	2069	49
Marathon	10 m	15.4 (3.3)	−4.8	−0.6	1905	2
	100 m^b^	–	–	–	–	–
	1 km	15.9 (3.6)	−4.3	−0.4	1752	2
	10 km	17.5 (4.7)	−2.7	0.6	1780	4
	100 km	20.2 (4.5)	0.0	0.9	2087	43
Wawa	10 m	16.3 (3.1)	−3.8	−0.8	1904	5
	100 m	16.4 (3.2)	−3.7	−0.7	1945	7
	1 km	16.9 (3.5)	−3.2	−0.5	1901	5
	10 km	17.9 (3.9)	−2.2	0.2	1930	11
	100 km	20.1 (5.0)	0.0	1.7	1937	40
Montreal River Harbour	10 m	18.3 (4.3)	−1.8	0.9	^b^	28
	100 m	18.3 (3.8)	−1.8	0.3	2128	33
	1 km	18.6 (4.0)	−1.5	0.4	2112	42
	10 km	18.7 (4.4)	−1.4	0.7	2161	18
	100 km	Same as Wawa 100 km site				

*Note*: The mean July daytime temperature and standard deviation are given. The difference between each logger and that transect's control site at 100 km is given, as well as the difference between shoreline sites and the nearest meteorological (met) station. Negative values refer to temperatures that are colder than reference sites. Growing degree days greater than 0°C (GDD_0_) and the average number of days above a 20°C threshold for boreal forest transition are given. Sites are listed geographically, from furthest west to furthest east.

^a^ GDD_0_ calculated from April 1, 2020–September 30, 2020.

^b^ Data missing due to lost temperature logger.

### 3.3 Buffering of temperatures

**FIGURE 4:**
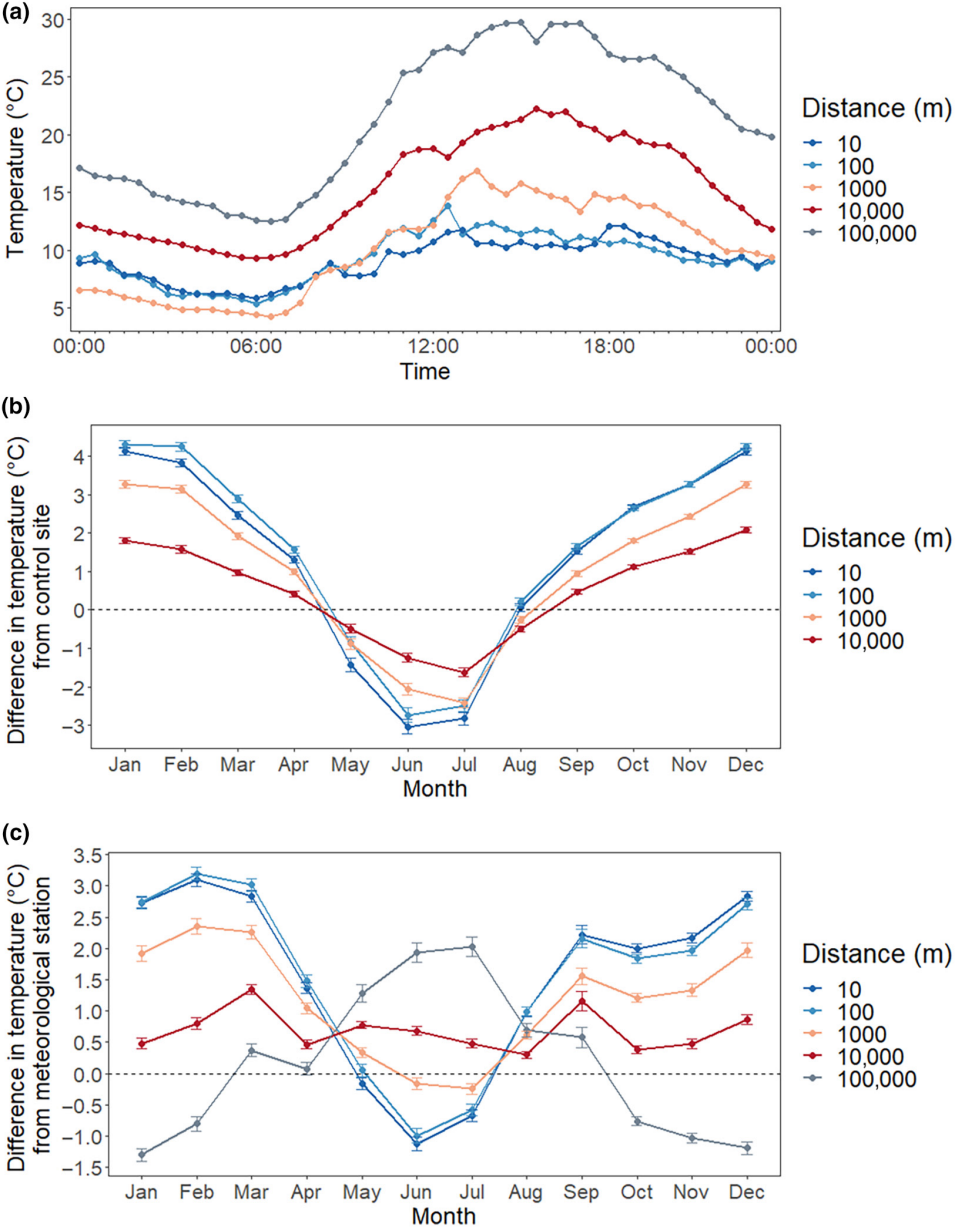
Temperature differences between distances and reference conditions. Daily raw temperature readings at Terrace Bay for the hottest day across our study period (July 2, 2019) (a) show daytime temperatures can be nearly 20°C cooler at nearshore sites. The monthly temperature difference between shore/inland sites and control sites (b) shows summer temperatures of up to 3.0°C cooler than control sites at 100 km. The mean annual temperature difference between shore/inland sites and the nearest meteorological station (c) shows summer temperatures up to 1.0°C cooler than the nearest station and up to 3.5°C warmer in winter. Positive values refer to temperatures that are warmer than control sites, and negative values refer to temperatures that are colder than control sites.

Shorelines (10 m) and immediately adjacent forests (100 m) showed the highest degree of buffering from reference site (100 km) temperatures. At Terrace Bay, daytime temperatures nearshore reach as high as 19.2°C cooler than reference sites (Figure 4a). For example, on the hottest recorded day within our study period (July 2, 2019), peak temperatures at the 100 km reference site averaged 29.5°C, while the 10 m site was only 10.3, nearly 20°C cooler. Inland sites were also much cooler during peak temperatures. The 100 m site was 18.9°C cooler, while the 1 km site was 14.0°C cooler, and the 10 km site was 8.4°C cooler. Mean monthly temperatures in winter were up to 4.3°C warmer at nearshore sites than at reference sites, and up to 3.0°C cooler in summer (Figure 4b). Sites further inland, at 1 and 10 km, still received a buffering effect from the lake, but the effect decreased with increasing distance from the lake.

Sites also showed buffering from the nearest meteorological station, although stations varied in their inland distances (3.75–10.27 km). On average, shoreline sites were 3.2°C warmer in winter than meteorological stations and 1.1°C cooler in summer (Figure 4c). Sites at 100 m showed the same trend, and sites at 1 km were up to 2.3°C warmer in winter but did not significantly differ from meteorological stations in summer. Sites at 10 km were similar to meteorological station temperatures, which were at the same general distance from the lakeshore, while inland reference sites at 100 km were up to 2.0°C warmer. Higher variability and temperature differentials at sites immediately adjacent to the shore highlight microclimate effects important for cold‐adapted species that are not reflected in standard available meteorological data.

### 3.4 Geographic trends in temperature differentials

The mean July daytime temperature at 100 km inland was not found to be significantly different across sites (*p* = .39), despite changes in latitude. Thunder Bay, on the western shore, had shoreline temperatures that were marginally significant from 100 km reference sites (*p* = .08). Sites further north that had more exposure to winds and deeper water offshore had shoreline temperatures that were significantly different from the 100 km distance. Terrace Bay and Marathon had the coldest July daytime temperatures, at 14.8 and 15.4°C, respectively (Figure 3). These sites also show the highest degree of buffering from inland sites, differing up to 5.8°C from 100 km reference sites. Despite being located on the exposed east shore but also being the furthest south transect, Montreal River Harbour had shoreline temperatures that were significantly different from inland temperatures (*p* = .003); however, average shoreline temperatures were only 1.8°C cooler than 100 km sites.

July daytime temperature buffering of sites from reference plots at 100 km varied geographically. Sites near Thunder Bay, on the west shore, showed little temperature change from the reference site. Similarly, sites at Montreal River Harbour differed little from reference plots (less than 2.0°C), despite the transects location on the exposed east shore. Wawa, on the exposed east shore and further north than Montreal River Harbour, showed moderate buffering from reference sites, with shore and inland sites differing by up to 3.8°C. Schreiber, Terrace Bay, and Marathon, at the most exposed and northernmost sites, show the highest degree of shoreline buffering, with 10 m sites 4.3, 4.8, and 5.8°C colder than reference sites, respectively. One hundred meter and 1 km sites at Terrace Bay and Marathon still remain greater than 4.0°C colder than reference sites, but 10 km sites warm to only 2.5 and 2.7°C colder. Refer to Table 1 for the temperature difference between the sites and their associated 100 km reference site.

### 3.5 Drivers of temperature buffering

July daytime temperature buffering between nearshore inland loggers and reference loggers was best predicted by elevation above the water (m), shore width (m), July water surface temperature (°C) within 10 km of shore, and depth of water (m) within 10 km of shore (*R* ^2^c = 0.19; Table 2). Specifically, the July daytime temperature difference is negatively related to elevation, with a 1 m gain in elevation resulting in a 0.11°C decrease in temperature difference. Shore width is positively related to temperature difference, with a 1 m increase in shore width resulting in a 0.09°C increase in temperature change. July water surface temperature had the greatest influence on temperature difference, with a 1°C increase in surface temperature resulting in a 0.85°C decrease in temperature difference. The depth of water within 10 km of the shore also negatively affects the temperature difference, with a 1 m decrease in depth (water getting shallower) resulting in a 0.07°C decrease in the temperature difference.

**TABLE 2:** Model output for drivers of temperature differentials.

Variable	*β*	SE	*z*	*p*
Elevation (m)	−.011	0.001	−7.277	<.001
Shore width (m)	.089	0.018	4.905	<.001
Water surface temperature (°C)	−.851	0.135	−6.302	<.001
Water depth (m) within 10 km	−.076	0.012	−6.455	<.001
Intercept	3.441	2.576	1.336	.182

*Note*: Variable name, estimated coefficient (*β*), standard error (SE), z statistic (*z*), and significance values (*p*) are given.

### 3.6 Growing season

**FIGURE 5:**
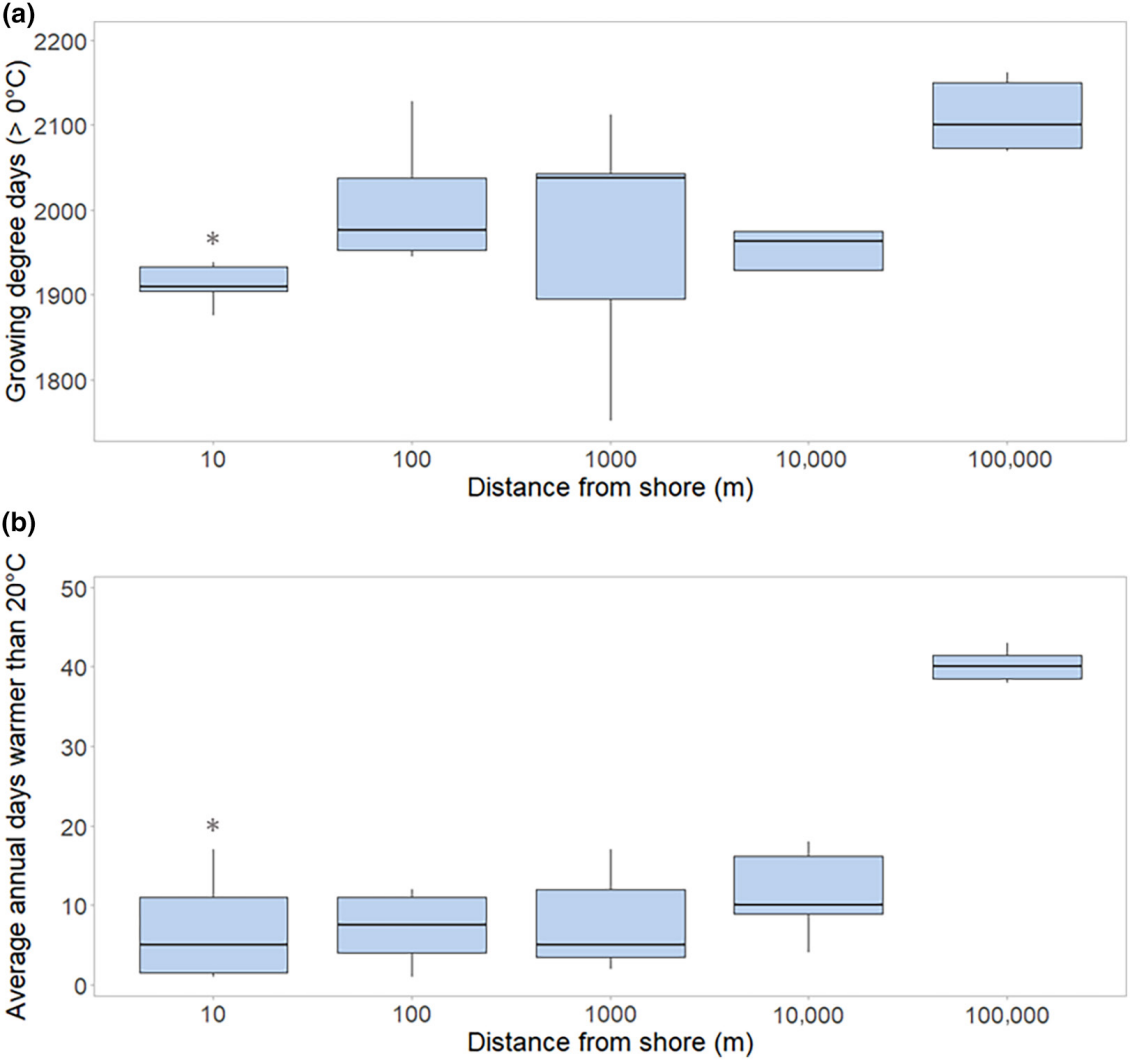
Differences in growing season indicators between sites. Growing degree days (GDD) greater than 0°C (a) were significantly lower at shoreline and inland sites than at control sites (100 km), reflecting shorter growing season conditions utilized by cold‐adapted species. Mean annual days warmer than 20°C (b) were reduced at all sites within 10 km of the shore, illustrating the importance of these sites for cold‐adapted species. A statistically significant difference is denoted with an asterisk (*).

Sites closer to shore had the shortest growing season (Figure 5a). At 10 m inland, growing degree days (GDD_0_, base 0°C) averaged 1950. Inland sites adjacent to the shore averaged 1972 GDD_0_ at 100 m, 1968 GDD_0_ at 1 km, and 2006 GDD_0_ at 10 km. Reference sites at 100 km averaged 2088 GDD_0_. Thus, shoreline sites were 138 GDD_0_ (7%) lower than references, illustrating the cooler temperatures and reduced growing season adjacent to the lake.

Sites 10 m from shore have the fewest number of days above 20°C, the threshold above which conifer‐dominated forests begin to transition to deciduous‐dominated forests (Figure 5b). Ten meter sites average 8 days per year, while 100 m, 1, and 10 km sites had similar average days above threshold at 11, 11, and 13 days, respectively. In contrast, reference sites at 100 km averaged 72 days above the threshold. Shoreline sites were variable, but some sites showed as few as 1 day above 20°C, illustrating the significantly cooler temperatures immediately adjacent to the lake.

Shoreline sites differed geographically in the number of days above the disjunct arctic–alpine plant threshold of 16°C. Thunder Bay had the greatest number of days, at 147. Despite being on the exposed eastern shore, Montreal River Harbour and Wawa showed 78 and 104 days above 16°C, respectively. Montreal River Harbour, however, was the furthest south of the sites, as well as being in deciduous forests. Rossport, Schreiber, and Marathon had 74, 60, and 70 days above 16°C; however, Terrace Bay had the fewest number of days, at 41.

### 3.7 Microclimatic drivers of disjunct arctic–alpine plant occurrence

**FIGURE 6:**
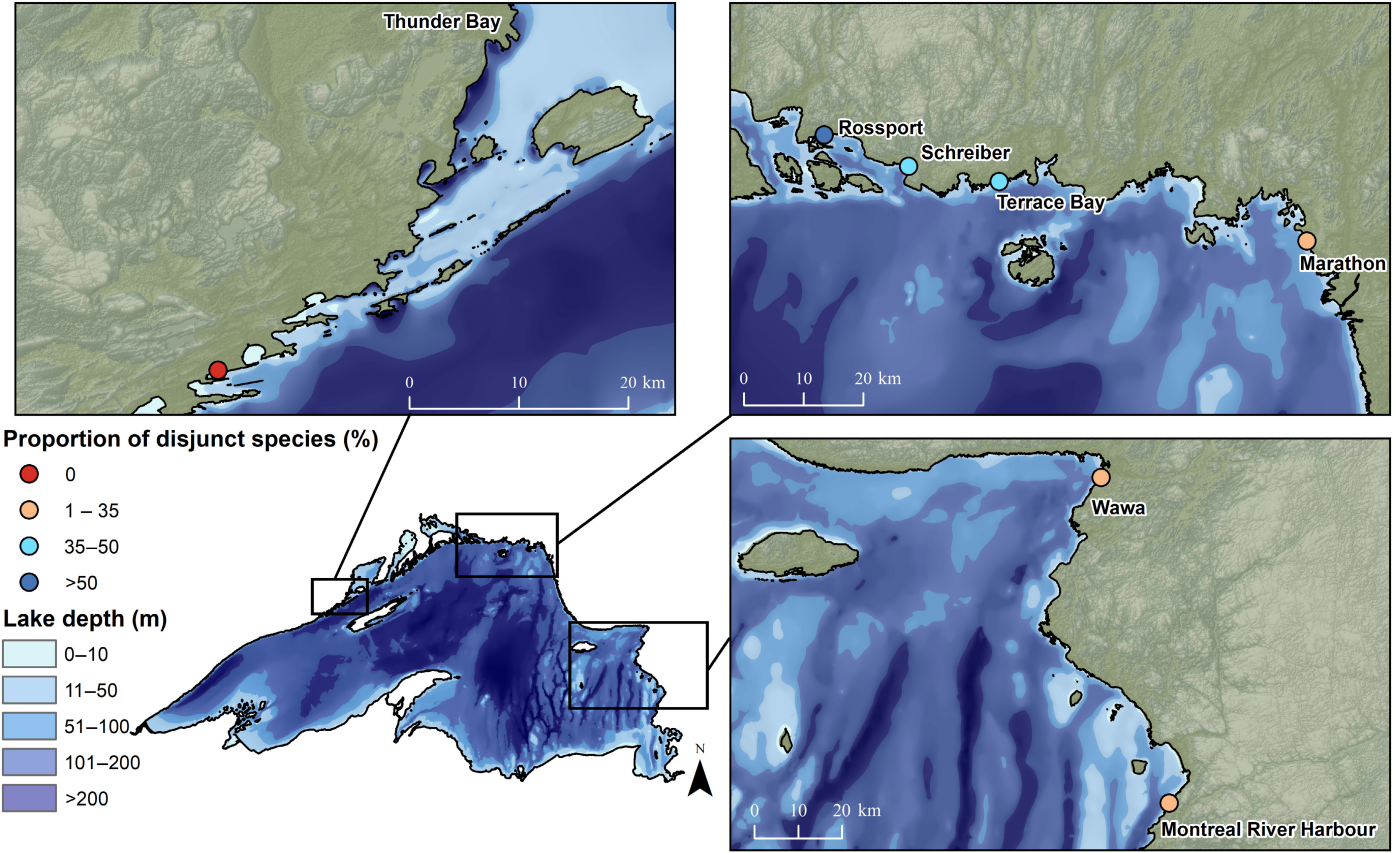
Geographic variation in proportion of disjunct arctic–alpine species at shore sites. The proportion (%) of disjunct species observed at each site is given. A higher proportion of disjunct species is driven by a shortened growing season (lower GDD_0_) and fewer days above a 16°C temperature threshold. The cold and exposed north‐central shore has the highest proportion of disjunct arctic–alpine species.

All sites had coverage values of 0.98 or higher, and so we used raw richness values as sites were assumed to be fully sampled (Chao et al., 2014). Patterns in proportion to disjunct species vary across the shoreline (Figure 6) and are best predicted by shore width (m) and the number of days per year above the threshold temperature of 16°C (*R* ^2^ = .85; Table 3). Specifically, a 1 m increase in shore width results in a 5.1% increase in the proportion of disjunct species. Average annual days above 16°C are negatively related to the proportion of disjunct species, with each additional day above 16°C resulting in a 2.7% decrease in the proportion of disjunct species.

**TABLE 3:** Model output for drivers of disjunct arctic–alpine plant occurrence.

Variable	*β*	SE	*z*	*p*
Shore width (m)	.051	0.006	8.328	<.001
Days above threshold temperature	−.027	0.001	−20.116	<.001
Intercept	.711	0.179	3.98	<.001

*Note*: Variable name, estimated coefficient (*β*), standard error (SE), z statistic (*z*), and significance values (*p*) are given.

## 4 DISCUSSION

We quantified the inland temperature gradient on the north shore of Lake Superior, determining the extent and degree of cooling provided by the lake effect. We also examined drivers of temperature buffering and described patterns in disjunct arctic–alpine plant occurrence at shoreline sites. Previous studies have described the general trends of the lake effect (Changnon & Jones, 1972; Scott & Huff, 1996) or have quantified the temperature gradient for a small part of the south shore (Hinkel & Nelson, 2012); however, to our knowledge, no other study has quantified the extent of shoreline and inland cooling, nor patterns in drivers of disjunct plant occurrence on the north shore, where climate refugia is most likely. In general, we found that while mean annual temperature did not significantly differ along an inland gradient due to offsetting buffering in winter and summer, there were strong seasonal effects closer to the lake. During the shoulder seasons of spring and fall, little difference was noted between sites as differentials between water and air temperatures were minimal. In the winter, due to the high specific heat capacity of water, solar radiation from the summer months is retained and released as the lake gradually cools over the winter. This results in warmer temperatures adjacent to the shoreline, with temperatures 6.5°C warmer than inland reference plots on average, but still winter (below freezing) conditions. While this winter warming phenomenon is well‐documented (Changnon & Jones, 1972; Scott & Huff, 1996), we do not discuss it further here as boreal and arctic/alpine plant species are dormant during this time, and thus warmer temperatures during this season do not contribute to the temperature buffering of the growing season.

Summer daytime temperatures, in particular July, show the largest temperature buffering at the shoreline. On average, shoreline sites were 3.1°C cooler than inland reference sites; however, this cooling effect varied, with some sites being up to 5.8°C cooler than reference sites, and on the hottest recorded day, up to 19.2°C cooler. Cold‐adapted boreal species are adapted to shorter growing seasons, and an earlier spring with warmer temperatures may put them at risk. Warmer, earlier springs result in premature leaf and flower development, making plant tissues vulnerable to late‐spring frost events (Ball et al., 2011). Advancing phenology may also create a phenological mismatch between flowering plants and their pollinators, resulting in a decrease in successful fruit production (Schmidt et al., 2016). Finally, cold‐adapted species are often sensitive to heat stress, with prolonged warm temperatures resulting in declines in productivity (Girardin et al., 2016; Kunert et al., 2022).

Length of growing season and threshold temperatures are important indicators of cold‐adapted species occurrence and persistence. Exposed shoreline sites in Terrace Bay and Marathon had 7.5% and 8.8% fewer GDD (1915 and 1905 GDD_0_, respectively) than at inland reference sites (2069 and 2087 GDD_0_). At these more exposed sites, reduced GDD can be observed up to 10 km inland, reflecting a shortened growing season for forests further inland as well. Similarly, shoreline sites and sites immediately adjacent to the shore had the fewest number of days above 16°C, a threshold that predicts the presence of arctic–alpine plants (Hillman & Nielsen, 2023). Above this threshold, arctic–alpine plants are less likely to be observed, and therefore sites with fewer days above 16°C reflect suitable habitat and growing conditions for these and other cold‐adapted plants. On average, sites 10 m inland had 67 days per year warmer than 16°C. Even at the shoreline, however, we observed high variability, with Terrace Bay having only 41 days per year warmer than 16°C, while Thunder Bay had 147 days per year over 16°C, on average. Similarly, individual transects varied in the number of days warmer than 20°C per year, a threshold at which boreal forest transitions to deciduous forest (Frelich et al., 2021). Sites on the north and northeast shores had 5 or fewer days per year warmer than 20°C, and cool temperatures at these sites extended 10 km inland, with inland sites having 10 or fewer days per year warmer than 20°C. This far‐inland cooling illustrates the significant cooling effect on exposed north and northeast shores and provides context for the buffering effect Lake Superior may have on forest transitions.

The magnitude of shoreline and inland cooling varies geographically, driven by changes in shoreline configuration and degree of exposure. The degree of temperature buffering is influenced by elevation, July water surface temperature within 10 km of shore, water depth within 10 km of shore, and shore width, which is a proxy for exposure. High winds produce large waves, and ice flows up onto shorelines, removing vegetation and preventing trees from establishing. Regions of the lake with wider shorelines thus receive higher wind and wave action, making them more exposed to the lake's weather. On sections of the shoreline that are protected by waves or on the west shore, which is upwind, trees are generally observed growing right to the lake's edge, with little or no exposed bedrock. Deeper water brings cooler temperatures to the surface, reflecting the cooler summer water temperatures and maintaining cooler temperatures inland. Colder shorelines therefore tend to be adjacent to the deepest parts of the lake. Lastly, changes in elevation are modest and have little effect on microclimate. Changes in elevation between sites are unlikely to be the primary driver of temperature differentials between shoreline and reference sites, as reference sites were only 266–285 m higher in elevation. Indeed, despite a modest increase in elevation and mostly higher latitude, reference sites were warmer in the summer. All shoreline and inland sites were cooler than their associated reference sites. The Terrace Bay shoreline differs in elevation from its reference site by only 145 m, but had the greatest temperature differences between the shoreline and reference sites.

Patterns in disjunct arctic–alpine plant occurrence reflect changes in growing season conditions across sites. The proportion of disjunct arctic–alpine plants at shoreline sites is related to growing degree days (GDD_0_) and the number of days per year warmer than 16°C. In general, sites that had fewer days warmer than 16°C and had the shortest amount of GDD_0_ showed the highest proportion of disjunct species on their shorelines. Rossport, Schreiber, and Terrace Bay, with the shortest growing season (GDD_0_ < 1940) and with fewer than 75 days per year warmer than 16°C (Terrace Bay had only 41), have the highest proportion of disjunct plants. Even sites with low overall species richness have a high proportion of disjuncts, suggesting that the habitat is preferred by disjunct arctic–alpine species over more common boreal species. Thunder Bay, which is warmer and has a higher GDD_0_, contains no disjunct species on its shoreline. These growing season indicators of disjunct plant occurrence reflect overall cooler conditions, as sections of the lake with deeper water, cooler water temperatures, and wider shorelines had a higher proportion of disjunct species. Marathon, one of the coolest sites, had a low proportion of disjunct species on its shoreline; however, exposure here is very high, and the available bedrock is worn away by wave action, which reduces the available microhabitat for plant growth. Sites that were highly exposed but were slightly above the lake level were more suitable habitat for disjunct species.

Variation across the lake can be attributed to differences in geographic location and latitude. Thunder Bay, on the far west shore, is at a lower latitude than Terrace Bay and Marathon, and being upwind of the lake, it does not receive the consistent cooling westerly winds that have crossed the widest and deepest parts of the lake. Wawa and Montreal River Harbour, while located on the highly exposed eastern shore, are further south in latitude and do not have as strong a cooling effect as sites further north. At Montreal River Harbour in particular, the conifer‐dominated forest begins to transition to deciduous forest, reflecting a warmer climate. Here, the cooling effect is not observed, with all sites along the inland gradient differing by less than 1°C. Rossport and Schreiber, located on the north shore, are at a higher latitude and do receive winds across the lake; however, their cooling effect is lower than both Terrace Bay and Marathon. Rossport, in particular, is protected by a series of islands and shallower waters that buffer high winds and waves and is generally warmer than other sites on the north shore. Indeed, the sheltering effect of the islands creates such protection from cold temperatures and waves that trees often grow right to the water's edge, removing the open bedrock habitat suitable for arctic–alpine disjuncts that is prevalent at more exposed sections of the north shore. Terrace Bay and Marathon, both highly exposed and receiving winds across the deepest part of the lake, show the highest temperature buffering. Temperature differentials here are much higher, with July temperatures being over 5°C cooler than inland reference sites. The colder temperatures and shortened growing seasons are reflected in the prevalence, abundance, and diversity of arctic–alpine species, and these areas are considered hotspots for arctic–alpine occurrence (Given & Soper, 1981; Hillman & Nielsen, 2023).

The variation in inland meteorological station distances results in inconsistent temperature data for shoreline and lake‐adjacent sites. At Lake Superior, meteorological stations that are several kilometers inland do not reflect the local cooling effects on the shoreline, particularly during the winter and summer months when shore sites can be 3°C different from meteorological station data. In July, the cool effects of the lake are not always reflected further inland, with shoreline sites recording temperatures 1°C cooler than meteorological stations in adjacent towns. While not a dramatic difference, the localized cooling effects of the lake on shoreline sites are not always reflected by inland temperature data, and thus more localized monitoring of shoreline and adjacent forest temperatures will better inform measures of refugia. Additionally, meteorological stations may not reflect microclimate effects occurring within forested areas. Generally, stations are placed in open areas and do not measure temperature phenomena under the tree canopy (De Frenne & Verheyen, 2016). For microclimate‐based assessments of refugia potential, localized temperature studies such as ours can illustrate conditions that are more biologically relevant to our focal species (Ashcroft, 2018). Targeted placement of microclimate sensors, such as within a conifer forest or adjacent to disjunct arctic–alpine plants, can provide us with more accurate species‐ or habitat‐specific temperature data with which to make management decisions.

Lake Superior serves as a model for the ability of large water bodies to buffer temperatures far inland, which has implications for the persistence of cold‐adapted plant species under warming temperatures. The high exposure of the north shore provides a strong cooling effect, as wind builds over the widest and deepest parts of the lake and brings the coldest temperatures and highest wave action to the north and northeast shores. Open bedrock habitats immediately adjacent to the lake are known to be cold enough to maintain disjunct populations of arctic–alpine plants (Given & Soper, 1981); however, an understanding of the temperature buffering in adjacent inland forests provides context for the extent of lake‐mediated cooling. The north shore of Lake Superior serves as a transition zone between deciduous hardwood forests on the south shore and boreal forests to the north. At the northernmost sections of the lake, forests are considered true boreal forests (Goldblum & Rigg, 2010), dominated by conifers such as balsam fir, white spruce, and black spruce; however, climate warming threatens these conifer‐dominated forests. Warmer temperatures are projected to shift forest zones north in the Great Lakes regions, resulting in an overall decline in conifer species (Walker et al., 2002). These models do not consider the cooling effect of Lake Superior, however. The maintenance of cool temperatures up to 10 km inland from the lake could provide a band of suitable habitat, allowing conifers to persist as the climate further from the lake is projected to transition to a deciduous‐dominated forest. Conifer trees in the region are economically important, and management of timber resources within climate refugia could allow for their continued harvest. Coniferous tree species in the boreal region are also ecologically important, creating an understory microclimate that buffers warm temperatures (De Frenne et al., 2021). Currently, large portions of Lake Superior's north shore are protected, with Lake Superior National Marine Conservation Area and Pukaskwa National Park protecting shoreline habitats. However, only Sleeping Giant Provincial Park, Neys Provincial Park, and Pukaskwa National Park provide protection for conifer‐dominated forests on the northeast shore, where the cooling effect is largest. Large portions of conifer forest within the cooling band of the lake remain unprotected. An understanding of the extent and magnitude of the temperature buffering can better guide protected area management and forest resource management within this cooling band as climate change continues to drive forest transitions.

## AUTHOR CONTRIBUTIONS

**Ashley Hillman:** Data curation (lead); formal analysis (lead); methodology (equal); visualization (lead); writing – original draft (lead). **Scott E. Nielsen:** Conceptualization (lead); methodology (lead); supervision (lead); writing – review and editing (lead).

## FUNDING INFORMATION

Funding was provided by the Natural Sciences and Engineering Research Council of Canada (grant RGPIN‐2019‐06040).

## CONFLICT OF INTEREST STATEMENT

The authors declare no conflict of interest.

## Data Availability

Data supporting the findings of this study are available on Dryad at: https://doi.org/10.5061/dryad.q83bk3jpc.

## APPENDIX 1

See Tables A1 and A2.

**TABLE A1:** Site information for temperature logger locations.

Transect	Distance	Latitude	Longitude	Start date	End date	Elevation above lake (m)	Canopy cover (%)	Dominant tree
Thunder Bay	10 m	48.020910	−89.544461	2018‐07‐13	2020‐07‐31	6	87.00	*Populus balsamifera*
	100 m	48.021877	−89.543060	2018‐07‐13	2020‐07‐31	10	81.00	*Abies balsamea*
	1 km	48.029704	−89.545551	2018‐07‐13	2020‐07‐31	13	82.50	*Picea glauca*
	10 km	48.102712	−89.600124	2018‐07‐13	2020‐07‐31	123	82.25	*Abies balsamea*
	100 km	48.651319	−90.497893	2018‐07‐13	2020‐07‐31	290	^a^	^a^
Rossport	10 m	48.838369	−87.487893	2019‐07‐13	2020‐07‐31	4	87.25	*Betula papyrifera*
	100 m	48.839178	−87.487874	2019‐07‐13	2020‐07‐31	16	80.25	*Abies balsamea*
	1 km	48.847347	−87.487885	2019‐07‐13	2020‐07‐31	99	87.75	*Picea mariana*
	10 km	48.873729	−87.349843	2019‐07‐13	2020‐07‐31	130	86.00	*Abies balsamea*
	100 km	49.677519	−87.020057	2019‐07‐13	2020‐07‐31	197	76.00	*Abies balsamea*
Schreiber	10 m	48.794993	−87.293278	2019‐07‐13	2020‐07‐31	9	86.00	*Picea glauca*
	100 m	48.795759	−87.292670	2019‐07‐13	2020‐07‐31	20	89.50	*Betula papyrifera*
	1 km	48.801152	−87.283577	2019‐07‐13	2020‐07‐31	79	87.00	*Abies balsamea*
	10 km	48.873729	−87.349843	2019‐07‐13	2020‐07‐31	130	86.00	*Abies balsamea*
	100 km	49.677519	−87.020057	2019‐07‐13	2020‐07‐31	197	76.00	*Abies balsamea*
Terrace Bay	10 m	48.775849	−87.085177	2018‐07‐13	2020‐07‐31	9	85.75	*Picea glauca*
	100 m	48.776713	−87.085097	2018‐07‐13	2020‐07‐31	27	88.25	*Betula papyrifera*
	1 km	48.784756	−87.084675	2018‐07‐13	2020‐07‐31	70	89.25	*Betula papyrifera*
	10 km	48.865603	−87.083366	2018‐07‐13	2020‐07‐31	132	84.50	*Abies balsamea*
	100 km	49.677519	−87.020057	2018‐07‐13	2020‐07‐31	197	76.00	*Abies balsamea*
Marathon	10 m	48.696877	−86.380555	2018‐07‐13	2020‐07‐31	9	81.00	*Abies balsamea*
	100 m	48.696896	−86.379209	2018‐07‐13	2020‐07‐31	11	82.50	*Betula papyrifera*
	1 km	48.696821	−86.367006	2018‐07‐13	2020‐07‐31	92	80.25	*Picea glauca*
	10 km	48.705307	−86.251938	2018‐07‐13	2020‐07‐31	65	61.50	*Abies balsamea*
	100 km	47.803164	−85.038727	2018‐07‐13	2020‐07‐31	269	84.25	*Abies balsamea*
Wawa	10 m	47.908671	−84.852320	2018‐07‐13	2020‐07‐31	11	^a^	*Picea glauca*
	100 m	47.908663	−84.850955	2018‐07‐13	2020‐07‐31	44	83.50	*Picea glauca*
	1 km	47.900539	−84.846220	2018‐07‐13	2020‐07‐31	90	84.75	*Abies balsamea*
	10 km	47.911858	−84.717493	2018‐07‐13	2020‐07‐31	90	86.25	*Abies balsamea*
	100 km	47.791939	−89.533216	2018‐07‐13	2020‐07‐31	269	83.00	*Picea glauca*
Montreal River Harbour	10 m	47.236955	−84.648359	2018‐07‐13	2019‐08‐06	4	80.00	*Abies balsamea*
	100 m	47.236621	−84.646960	2018‐07‐13	2020‐07‐31	21	85.25	*Pinus strobus*
	1 km	47.239603	−84.625395	2018‐07‐13	2020‐07‐31	74	86.50	*Abies balsamea*
	10 km	47.240262	−84.587680	2018‐07‐13	2020‐07‐31	74	88.25	*Abies balsamea*
	100 km	47.791939	−89.533216	2018‐07‐13	2020‐07‐31	269	83.00	*Picea glauca*

*Note*: The location of sites is provided, as well as start and end date of data collection. Site characteristics of elevation above the lake surface (m), tree canopy cover (%), and dominant tree species observed are given. Sites are listed geographically, from furthest west to furthest east.

^a^ Data unavailable.

**TABLE A2:** Shoreline disjunct arctic–alpine species information.

Transect	Shore width (m)	Total species richness	Disjunct species richness	Proportion disjunct (%)	Disjunct species
Thunder Bay	2	12	0	0	NA
Rossport	11.3	3	2	67	*Poa glauca*
					*Trisetum spicatum*
Schreiber	24	10	5	50	*Pinguicula vulgaris*
					*Primula mistassinica*
					*Sagina nodosa*
					*Trichophorum cespitosum*
					*Trisetum spicatum*
Terrace Bay	14.7	20	8	40	*Hupperzia appressa*
					*Pinguicula vulgaris*
					*Primula mistassinica*
					*Saxifraga paniculata*
					*Solidago multiradiata*
					*Solidago simplex*
					*Trichophorum cespitosum*
					*Vaccinium uliginosum*
Marathon	17.7	3	1	33	*Calamagrostis purpurascens*
Wawa	21.3	11	3	27	*Primula mistassinica*
					*Solidago multiradiata*
					*Trichophorum cespitosum*
Montreal River Harbour	18.6	3	1	33	*Trisetum spicatum*

*Note*: Shore width (m) and species richness information for surveyed shoreline sites are provided. Total species richness, disjunct species richness, and proportion of disjunct species (%) at each site are given, and observed disjunct arctic–alpine species are listed for each site. Sites are listed geographically, from furthest west to furthest east.
